# Predictive modeling for step II therapy response in periodontitis - model development and validation

**DOI:** 10.1038/s41746-025-01828-3

**Published:** 2025-07-15

**Authors:** Elias Walter, Tobias Brock, Pierre Lahoud, Nils Werner, Felix Czaja, Antonin Tichy, Caspar Bumm, Andreas Bender, Ana Castro, Wim Teughels, Falk Schwendicke, Matthias Folwaczny

**Affiliations:** 1 Department of Conservative Dentistry and Periodontology, University Hospital, LMU Munich, GoethestraSSe 70, Munich, Bavaria Germany; 2 Department of Statistics, LMU Munich, Munich, Bavaria Germany; 3 Division of Periodontology & Oral Microbiology, Department of Oral Health Sciences, University Hospitals Leuven, KU Leuven, Leuven, Belgium; 4 OMFS-IMPATH Research Group, Department of Imaging and Pathology, University Hospitals Leuven, KU Leuven, Leuven, Belgium; 5 Munich Center for Machine Learning (MCML), Munich, Bavaria Germany; 6 Faculty of Dentistry, Department of Periodontology, Chulalongkorn University, Bangkok, Thailand

**Keywords:** Periodontitis, Outcomes research

## Abstract

Steps I and II periodontal therapy is the first-line treatment for periodontal disease, but has varying success. This study aimed to develop machine learning models to predict changes in periodontal probing depth (PPD) after step II therapy using patient-, tooth-, and site-specific clinical covariates. Models accurately predicted that healthy sites stay healthy, but performed suboptimally for diseased sites. Tuning improved performance, with PPD, tooth-site, and tooth-type identified as key predictors. Pocket closure was predicted with fair accuracy, with baseline PPD as the most relevant covariate. Models predicted improving pockets well but underperformed for non-responding sites, with antibiotic treatment and tooth type being the most influential features. While predictive performance for step II periodontal therapy based on routine clinical data remains limited, models can stratify periodontal sites into meaningful categories and estimate the probability of pocket improvement. They provide a foundation for site-specific outcome prediction and may support patient communication and expectations.

## Introduction

Assigning a prognosis for a disease or predicting therapy outcomes is a key step in clinical decision-making and precision medicine. It enables the differentiation between non-responding and responding patients along with individual adjustment of the treatment plan by advanced statistical methods. However, this approach remains to be adapted to periodontitis^1,2^.

Periodontitis is a highly prevalent chronic inflammatory disease, with its severe form affecting 9.8% of people worldwide^3,4^ and is believed to affect up to 50% of individuals in older age groups^5^. It leads to the destruction of tooth supporting tissues including bone loss, and is ultimately one of the main reasons for tooth loss in the industrialized world. This destruction is clinically reflected by the manifestation of periodontal pockets, which progressively increase in depth with growing disease severity. By controlling the dysbiotic subgingival biofilm and restoring symbiotic homeostasis within the subgingival periodontal microbiome, periodontal therapy aims to reduce periodontal inflammation and further progression of the disease^6^. Modern periodontal treatment follows a standardized schedule and is commonly carried out in four distinct and interrelated steps with different levels of invasiveness. The initial two phases constitute the first-line therapy, regardless of the disease’s severity^7^.

Step I periodontal treatment guides patient behavior change by informing, training, and motivating the patient toward improved control of primary and secondary risk factors for periodontitis, i.e., quality of routine oral hygiene measures as well as tooth-related plaque retention areas, smoking, or diabetes. Step II focuses on control of the subgingival biofilm primarily by application of systematic subgingival instrumentation. These first steps should always be carried out regardless of the stage of the disease. The mean reduction in periodontal probing depth (PPD), which is commonly used as a surrogate parameter for periodontal healing, ranges between 1.2–2.4 mm^8–10^. Commonly, the individual response of both steps is highly variable at patient-, tooth-, and site-specific levels. This variability, in turn, determines the need for costly follow-up treatments, either progressing to Step III, which includes surgical intervention, or to Step IV, non-surgical supportive periodontal care (SPC).

Numerous confounding factors on patient-, tooth- and site-specific levels have been identified to explain the variability in the individual response to periodontal therapy. Most studies in this field used classical statistical models to define particular subgroups by stratification, and reported on group-related associations rather than on true prognosis toward therapy outcomes^9,11,12^. Consequently, these studies were not fully able to delineate the complex multi-factorial etiology of the disease with multifaceted phenotypic clinical features, or to provide useful prediction models for clinical care.

Predictive modeling refers to analyzing data and identifying hidden patterns to predict event outcomes. Machine learning models try to learn these patterns automatically by minimizing the dissimilarity of the input to the output and optimizing the result iteratively by adjusting weights^13^. Previous studies have used machine learning to predict periodontal tooth loss, but given tooth loss being a rare event, the resulting models have not reached clinical usefulness^14,15^.

**Fig. 1:**
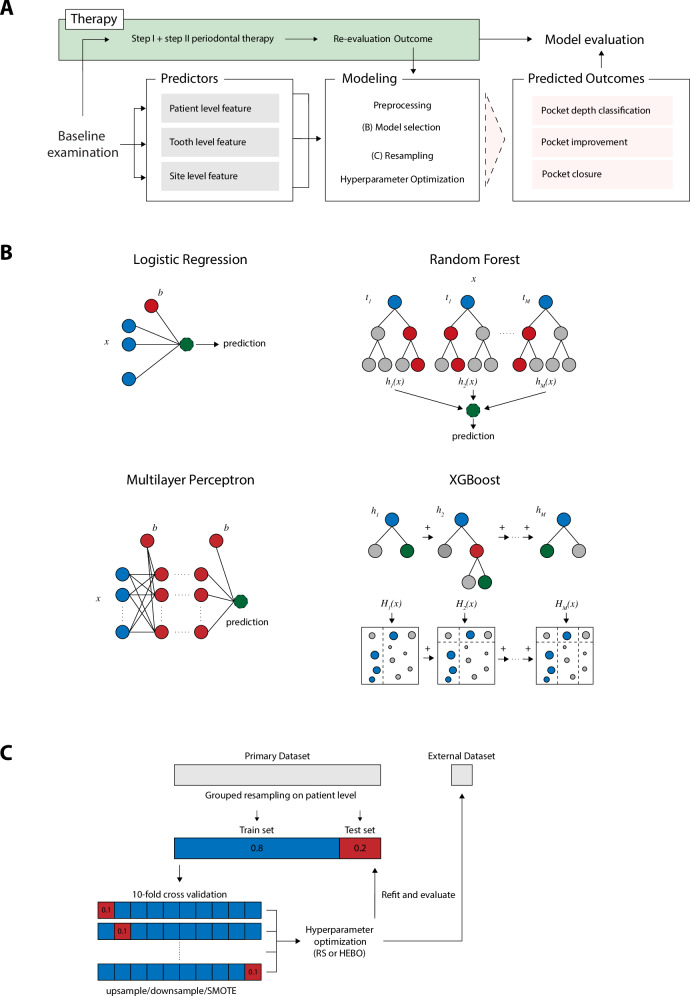
Overview of predictive modeling pipeline for periodontal therapy outcome prediction. **A** Schematic representation of the modeling pipeline. **B** Selection and structure of linear (left) and tree-based (right) predictive models used for outcome prediction. **C** Schematic of resampling strategy on patient level with a 80-20 train test split and ten-fold cross-validation. Hyperparameters were optimized by random search (RS) or Heteroscadistic evolutionary Bayesian optimization (HEBO). Models were internally validated on the independet test dataset and the external dataset.

Comprehensive predictions of short-term surrogate endpoints for periodontal therapy might be more beneficial from a clinical perspective but have not been adequately considered yet. This study aims to assess whether state-of-the-art machine learning models can predict the short-term outcomes of Steps I and II periodontal therapy based on routinely collected patient-specific, tooth-specific, and site-specific clinical parameters. Additionally, a predictive modeling pipeline tailored to periodontal therapy was developed, emphasizing ease of use and adaptability for future clinical as well as research applications (Fig. 1).

## Results

### Multiclass classification of PPD categories

**Fig. 2:**
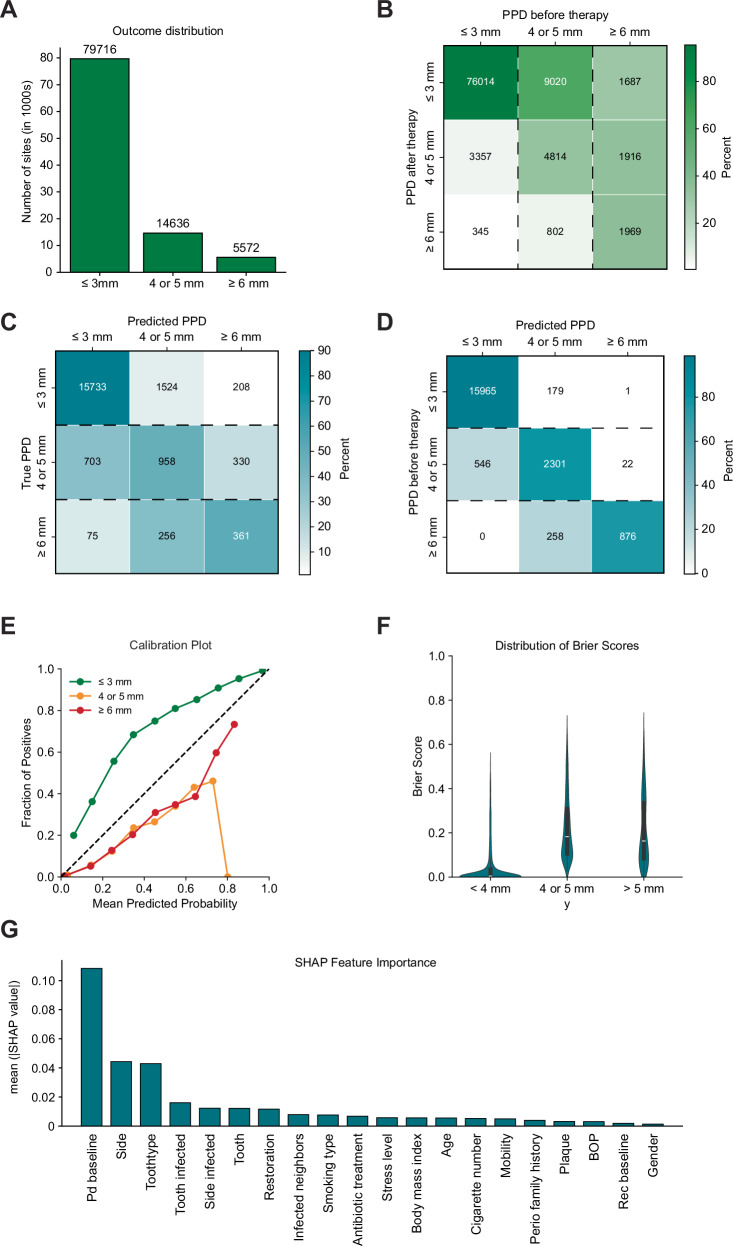
Multi-class prediction of categorical pocket depth after step II periodontal therapy by a random forest model tuned on F1 score. **A** Outcome distribution of categorical periodontal probing depth (PPD) post-therapy. **B** Confusion matrix showing changes in PPD before and after therapy in the dataset (normalized vertically). **C** Representative confusion matrix of predictions made by the best-performing model compared to the true outcome and **D** comparison of predictions to baseline PPD (normalized horizontally). **E** Calibration plot illustrating the agreement between predicted probabilities and observed outcomes. **F** Distribution of Brier scores for each category and **G SHapley Additive exPlanation** (SHAP) feature importance highlighting key predictors influencing the models predictions.

Descriptive analysis of the pocket depth distribution before therapy shows an imbalanced data distribution in the dataset with 79,716 in category 1 (PPD ≤ 3 mm), 14,638 sites in category 2 (PPD 4–5 mm), and 5572 in category 3 (PPD ≥ 6 mm) (Fig. 2A). A similar distribution was present in the external dataset. To address this imbalance, model benchmarking was performed using upsampling, downsampling, or the synthetic minority oversampling technique. The confusion matrix highlights class changes from before and after therapy (Fig. 2B). Most healthy sites stayed healthy (95%). ~60% of pockets in category 2 improved to category 1, while less than 10% of pockets changed from category 1 to category 2. Furthermore, 66% of category 3 pockets showed improvement, transitioning to either category 2 or 1 with similar extents (Fig. 2B).

The dummy classifier control shows a F1_macro_ score of 0.31. With the inclusion of features the performance improves in untuned logistic regression (LR) and random forest (RF) controls (Table 1). A total of 120 models were trained using different parameter combinations. Among these, tree-based models demonstrated the best performance, with a RF optimized for F1_macro_ and an eXtreme gradient boosting (XGBoost) model optimized for Brier Score yielding the best results (Table 1). On the one hand, overall accuracy decreased slightly by training to improve either F1 or Brier Score, while on the other, class-specific F1 scores improved for diseased pockets (category 1: 0.27–0.38; category 2: 0.30–0.41) (Fig. 2D, Table 1). To visualize predictions and performances, confusion matrices were generated for the best performing model (Fig. 2C). Comparison of baseline PPD to class prediction shows that the model predicted healthy sites stay healthy and approximately 30% of pockets improved from categories 2 or 3 to the respective better class, resulting in the aforementioned overall metrics. Thereby, the model predicted with a systematic underestimation of healthy sites and overestimation of diseased sites, which is demonstrated by the calibration plot (Fig. 2E). The most relevant features considered by the model were baseline PPD, tooth-site-location as well as tooth-type, while bleeding on probing (BOP), baseline recession, as well as gender were least relevant (Fig. 2G).

**Table 1:** Performance metrics of the best-performing models for multiclass classification of pocket depth, evaluated on the internal test set during development and validation on the external dataset

Phase	Model	Criterion	F1_macro_	Accuracy	Class F1 scores	Brier score
Control	Dummy classifier	Untuned	0.31	0.87	[0.93, 0.00, 0.00]	0.12
	LR	Untuned	0.52	0.88	[0.94, 0.32, 0.31]	0.08
	RF	Untuned	0.51	0.88	[0.94, 0.28, 0.31]	0.09
Development	Tuned RFt	F1_macro_	0.58	0.85	[0.92, 0.40, 0.42]	0.11
	Tuned XGBoost	Brier score	0.53	0.78	[0.87, 0.40, 0.31]	0.14
Validation	Tuned RF	F1_macro_	0.55	0.80	[0.89, 0.43, 0.33]	0.14
	Tuned XGBoost	Brier score	0.52	0.81	[0.90, 0.43, 0.22]	0.13

Applying the models to the external dataset yielded performance comparable to the internal test set, with a slight decline observed in category 1 and a significant decline in category 3. The Brier score increased marginally compared to the development model, suggesting a reduction in predictive certainty. In conclusion, while the model performs sub-optimally for baseline-affected sites, model performance can be improved by inclusion of features and training, thereby categorizing periodontal sites into meaningful categories.

### Binary classification of pocket depth improvement

**Fig. 3:**
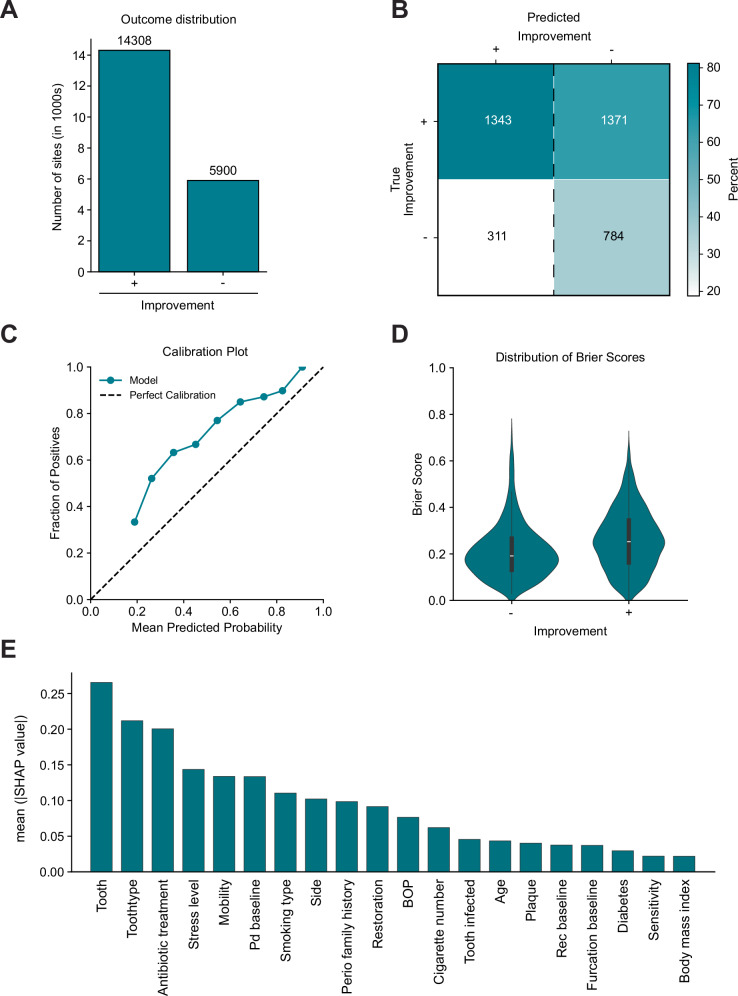
Prediction of pocket improvement after step II periodontal therapy by a logistic regression trained on F1 score. **A** Outcome distribution of pocket improvement in the complete dataset. **B** Confusion matrix of predictions made by the model on the test-dataset compared to the true outcome (normalized vertically). **C** Calibration plot illustrating the agreement between predicted probabilities and observed outcomes. **D** Distribution of Brier scores for each category and **E** SHapley Additive exPlanation (SHAP) feature importance highlighting key predictors influencing the models predictions.

For this analysis, only sites with PPD > 3 mm were included, resulting in a dataset of 20,280 sites. Among these, 71% showed improvement, while 29% failed to improve (Fig. 3A). Therefore, a dummy classifier achieved an accuracy of 0.71 by predicting that all sites improve (Table 2). Incorporating features into untuned LR and RF models resulted in similar overall accuracies but with different predictions, yielding slightly higher F1 scores compared to the dummy classifier. Brier scores increased and indicated relative uncertainty in the models’ probabilistic predictions. Hyperparameter tuning, based on either the F1 score or the Brier score, improved the targeted metric and to a lesser extent also the other metric (Table 2). Despite these optimizations, both metrics remained suboptimal, with Brier scores of 0.19 and 0.24, and F1 scores of 0.45 and 0.10, respectively (Table 2). Notably, the F1-tuned model achieved a recall of 0.63 and a negative predictive value of 0.81, indicating its effectiveness in identifying sites likely to improve. However, its precision for predicting non-improvement was low (0.35), suggesting difficulty to accurately predict non-responding sites (Fig. 3B, Table 2). This was further supported by the calibration plot, which revealed a tendency to underestimate the probability of improvement (Fig. 3C). Additionally, the Brier score distribution highlighted the relative uncertainty in predicting both improvement and non-improvement outcomes (Fig. 3D). Feature importance analysis identified the specific tooth, tooth type, and antibiotic treatment as the most significant predictors (Fig. 3E). In the validation dataset the models overall performance dropped slightly (ROC AUC: 0.62). The model showed better recall (0.77) but decreased precision (0.1) and F1-score (0.26) (Table 2).

**Table 2:** Performance metrics of the best-performing models for pocket depth improvement, evaluated on the internal test set during development and validation on the external dataset

Phase	Model	Criterion	F1	Precision	Recall	Accuracy	Brier Score	ROC AUC
Control	Dummy classifier	untuned	0.0	0.0	0.0	0.71	0.20	0.50
	LR	Untuned	0.06	0.62	0.03	0.72	0.29	0.63
	RF	Untuned	0.08	0.35	0.04	0.70	0.20	0.61
Development	Tuned LR	F1	0.45	0.35	0.63	0.58	0.24	0.63
	Tuned LR	Brier score	0.10	0.48	0.06	0.72	0.19	0.63
Validation	Tuned LR	F1	0.26	0.16	0.77	0.43	0.28	0.62
	Tuned LR	Brier score	0.08	0.19	0.05	0.85	0.15	0.61

In summary, while the prediction of pocket improvement based on baseline clinical data demonstrates suboptimal overall performance, pockets predicted to improve were very likely to improve clinically.

### Binary classification of pocket closure

**Fig. 4:**
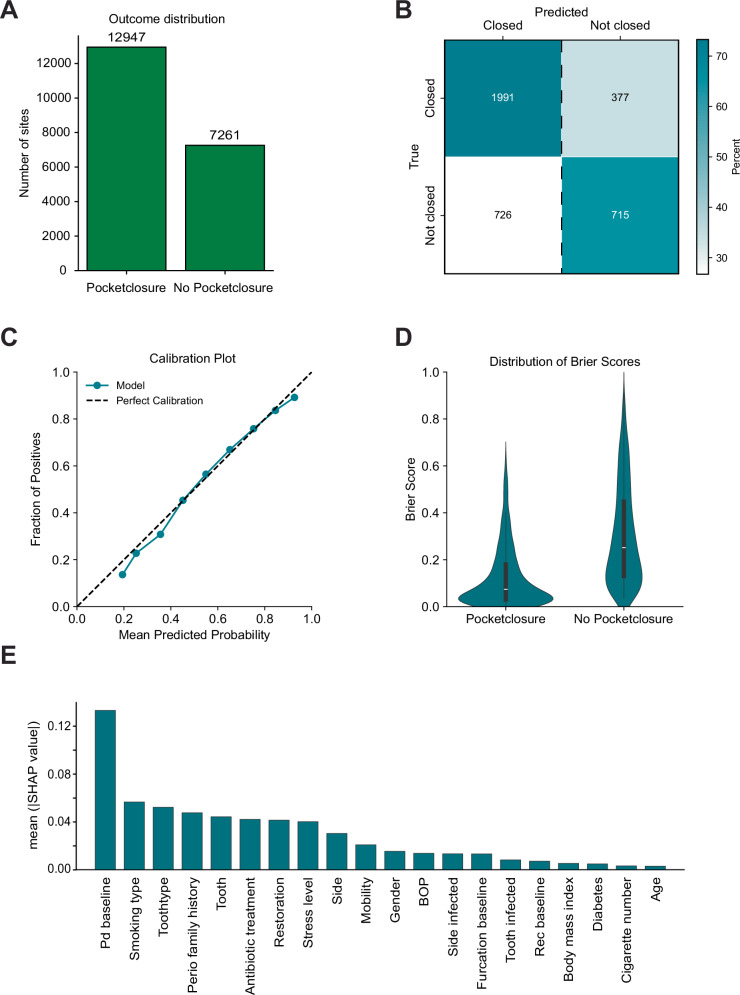
Prediction of pocket closure after step II periodontal therapy by a Multilayer Perceptron trained on Brier Score. **A** Outcome distribution of pocket closure in the complete dataset. **B** Confusion matrix of predictions made by the model on the test-dataset compared to the true outcomes (normalized vertically). **C** Calibration plot illustrating the agreement between predicted probabilities and observed outcomes. **D** Distribution of Brier scores for each category and **E** SHapley Additive exPlanation (SHAP) feature importance highlighting key predictors influencing the models predictions.

Similar to the improvement model, the dataset comprised 20,208 pockets, with an outcome distribution of 62% of pockets closing after step II therapy and 38% remaining unclosed (Fig. 4A). The inclusion of baseline clinical features improved prediction accuracy, with LR and RF models achieving accuracies of 71% and 70%, respectively, and ROC AUC values of 0.74 and 0.61 (Table 3). Training models further enhanced performance, albeit modestly. Among these, a MLP model optimized for Brier score achieved the highest accuracy (0.72) (Fig. 4B) and the best Brier score (0.19). The model demonstrated near-perfect calibration (Fig. 4C). Notably, pockets predicted to close exhibited significantly better Brier scores (median = 0.08) compared to those predicted to remain unclosed (median = 0.25) (Fig. 4D). SHAP feature analysis revealed that the model primarily relied on baseline PPD, with additional contributions from other covariates such as smoking habits, tooth type, family history of periodontitis, the specific tooth and systemic antibiotic treatment (Fig. 4E).

**Table 3:** Performance metrics of the best-performing models for pocket closure, evaluated on the internal test set during development and validation on the external dataset

Phase	Model	Criterion	F1	Precision	Recall	Accuracy	Brier Score	ROC AUC
Control	Dummy classifier	Untuned	0.00	0.00	0.00	0.62	0.24	0.50
	LR	Untuned	0.52	0.68	0.42	0.71	0.20	0.74
	RF	Untuned	0.08	0.35	0.04	0.70	0.20	0.61
Development	Tuned LR	F1	0.61	0.55	0.67	0.69	0.21	0.74
	Tuned multilayer perceptron	Brier score	0.55	0.64	0.49	0.72	0.19	0.74
Validation	Tuned LR	F1	0.54	0.52	0.57	0.88	0.16	0.88
	Tuned multilayer perceptron	Brier score	0.44	0.64	0.34	0.76	0.16	0.77

Models showed very good discrimination (ROC AUC = 0.88) in the external validation dataset with very good confidence in predictions as indicated by the Brier score.

## Discussion

Prediction modeling in periodontology has largely been unsuccessful, primarily due to its focus on long-term outcomes with tooth loss as the therapeutic endpoint and mean reported *c*-statistics of 0.67^1^. One major challenge is the extended follow-up period, often exceeding 10 years, which introduces various sources of uncertainty and bias^1,2^. Dropout rates at longer observation periods lead to incomplete data sets, potentially skewing the entire analysis. Additionally, patients are commonly enrolled into routine SPC with heterogeneous rigor based on disease severity and dynamics causing relevant prognostic uncertainties, that might further impair predictive modeling^7^. To overcome these issues, short-term prediction of periodontal therapy outcomes may be more feasible. Shorter follow-up periods facilitate data collection, reduce attrition bias, and allow for more standardized comparisons of treatment efficacy. By focusing on discrete steps within the periodontal therapy schedule rather than on ultimate long-term outcomes, i.e., tooth loss, predictive models may achieve greater accuracy and clinical applicability. Accordingly, the present analysis specifically considered the initial therapy steps of periodontitis. Furthermore, the surrogate endpoints, such as PPD change/improvement, PPD categorization or pocket closure reflect therapy response as well as inform on disease progression comprehensively with a more fine-grained discrimination than tooth loss^10,16^. Our findings suggest that prediction modeling based solely on basic clinical features at the site level yielded limited performance. However, they also demonstrated that machine learning algorithms can enhance the prediction of therapy outcomes for individual periodontal pockets, laying the foundation for probabilistic outcome prediction.

### Performance of predictive models and limitations

The pocket classification model demonstrated high overall accuracy. However, it has to be taken into account that this is mostly determined by healthy sites, which are clinically less relevant. This is evident by comparison with baseline models as well as a more nuanced performance distinction. Such distinctions have often been overlooked in previous periodontal prediction models, highlighting the limitations of past studies and underscoring the need for rigorous model evaluation^1,2^. Here, however, both tuning criteria improved the classification of diseased sites while performance for healthy sites declined slightly, suggesting that machine learning models can enhance the prediction of clinically relevant cases. The responsible predictors are also statistically and clinically feasible. The predictive value of baseline PPD is a direct correlation to disease progression and provides a foundational basis from which a general classification of each site is performed. Additionally, anatomical site and tooth type have been associated with step II therapy response in the past,^9,17,18^ but they have not yet been evaluated properly for their predictive potential. Clinically, anterior teeth are less often affected by periodontitis, are easier to clean by daily oral hygiene and provide better access than molars in step II therapy^19–21^. It has also been demonstrated that long-term predictions for anterior teeth are more accurate, while improvements in molars are less predictable^22^. This also holds true for oral and vestibular sites compared to approximal sites^11,12^. The predictive relevance of anatomical locations clearly emphasizes the clinical significance of site-specific outcome prediction, taking into account various patient- and tooth-level features. In fact, almost 80% of the relevant factors determining the periodontal treatment success were site-specific factors before^12^.

The ROC AUC of 0.64 and the Brier score distribution in the improvement model underscore the challenge to predict changes in pocket depth at diseased sites based solely on baseline clinical data. Notably, optimizing for F1-score introduces a recall bias, yielding a model that accurately identifies improving sites but not non-improving sites. This bias is evident in the calibration plot, where non-improvement is systematically overestimated, particularly at lower predicted probabilities. The relatively high but more evenly distributed SHAP values in the improvement model suggest that multiple factors contribute to therapy outcomes, with no single dominant predictor. This finding reflects not only the multi-factorial nature of periodontitis^6^ but also considerable statistical uncertainty. Similar to the pocket classification model, the specific tooth and tooth type are the key determinants in predicting therapeutic success, followed by adjunctive systemic antibiotic treatment. While antibiotics enhance periodontal therapy efficacy, they are typically indicated for more severe cases in younger patients, introducing potential confounding. Notably, tooth mobility, previously linked to poorer outcomes in non-surgical intervention,^11^ was not significantly associated with PPD reduction in this cohort when analyzed with conventional multivariate statistical methods^9^. However, in prediction modeling, it acted as a key predictor in the improvement model, highlighting potential limitations of classical association analyses and the complementary insights provided by advanced predictive techniques.

Pocket closure has been defined as a surrogate endpoint of periodontal therapy because shallow pockets have been shown to be less susceptible to disease recurrence and progression^16^. The trained models were able to reliably discriminate between outcomes with perfect calibration, even though discrimination in the improvement model was suboptimal. Accordingly, the most relevant prognostic feature as found herein was the baseline PPD. The second most important determinant for treatment success was smoking in the current analysis, which is in line with numerous previous studies indicating smoking as a risk factor for periodontitis^23,24^ that has a considerable negative impact on the rate of pocket closure^17,25^. Other features were closely corresponding to the other models.

Interestingly, furcation involvement (FI) and diabetes showed only weak predictive power in all models, despite both factors being associated with reduced therapy response in this dataset^9^ and in other studies^19^. These contradictory results may be attributed to the limited number of cases and, therefore, imbalanced expression of both factors in the dataset and the quality of the data itself. Only 10% of patients had a diagnosis of diabetes, which may have attenuated its predictive impact. Partially in line, albeit FI was present in 42% of tooth surfaces, a lot of teeth anatomically cannot develop any FI, potentially masking its predictive potential. The sample size of the dataset might be insufficient to learn these feature connections in complex models reliably. Apart from further increasing the sample size, the inclusion of data from various centers for external validation might be appropriate strategies to further improve model performance and its generalizability. Furthermore, a diagnosis of diabetes alone does not reflect the level of glycemic control or the current status of the disease. The predictive potential could be enhanced by incorporating a more fine-grained discrimination and objective measures such as hemoglobin A1c, which would allow for a more comprehensive classification of the glycemic state in individuals with diabetes.

External validation is essential for assessing the generalizability of predictive models. As expected, a slight decline in performance was observed here. However, discrimination performance remained stable, with a slight decrease in the PPD categorization and improvement model. Notably, the pocket closure model demonstrated enhanced discrimination and improved Brier scores. The validation dataset was derived from a secondary care setting, whereas the training data originated from primary care. The validation cohort included a higher proportion of stage III patients with generalized periodontitis (Supplementary Table 1), but interestingly also a higher proportion of pockets showing improvement and achieving closure. This suggests that non-surgical periodontal treatment outcomes in specialist settings may be more predictable, possibly due to greater practitioner expertise, differences in patient selection and compliance, or other setting-specific factors. Importantly, this also indicates that the models may be applicable across different clinical environments.

This also leads to this study’s limitation. Only routinely collected standard clinical data have been included in modeling, which may not reflect all relevant determinants for periodontal disease progression and treatment outcomes. Moreover, future studies should consider incorporating objective measures of practitioner experience and treatment efficacy, such as subgingival plaque removal efficiency, to better account for the true therapy quality. Additionally, it is commonly accepted that periodontitis is a complex multi-factorial disease influenced not only by clinical parameters but also by numerous intrinsic factors, particularly host genetic and microbiological factors^16^. For instance, the IL-6-174 G/C polymorphism has been linked to a reduced treatment response to non-surgical subgingival debridement^12^. Incorporating biomarkers, such as matrix metalloprotease or interleukin levels, could enhance predictive accuracy^26^. Objective behavioral data, such as oral hygiene habits recorded via electronic smart toothbrushes before and after Step I periodontal therapy, may further refine model performance^27^. In addition, soft feature imputation could be particularly relevant for periodontitis by establishing connections between individual sites, teeth, and the overall patient. For example, incorporating patient-level plaque index alongside site-specific plaque occurrence could provide a more comprehensive representation of both general oral hygiene behavior and local predisposing factors. Similarly, combining the overall progression of periodontitis, based on stage and grade, with individual PPD per site would allow for a more comprehensive inclusion of disease severity.

Lastly, even though periodontal charting in itself is standardized and always performed in periodontal therapy, it is performed manually, which can result in reporting errors and in turn lead to reduced data quality. Incorporation of more objective data collection methods independent of examiners’ individual reliability, which includes standardized imaging modalities such as radiographs, surface scans, or even multi-modal approaches, might not only improve data quality but also provide a better and more complete picture of the patient.

### Clinical outlook

Clinically, the decision for SPC is generally decided upon after reevaluation of step II therapy. Therefore, these models may initially not appear to contribute significantly to treatment decision-making. However, oral hygiene instruction and patient education are central steps in periodontal therapy^7^. Predictive models together with appropriate visualizations might help to improve these steps and enhance patient motivation^28^. The pocket closure model provides valuable insights into the expected surrogate endpoint of therapy, offering a clearer understanding of the potential outcome for each tooth site. In parallel, classification into pocket categories can be used to communicate the possibility of requiring step III periodontal therapy, specifically surgical treatment of teeth affected by severe periodontitis; potentially motivating patients to engage more actively in improving modifiable risk factors such as their daily oral hygiene or smoking habits during Step II therapy. Finally, by reclassifying sites with ’no improvement’ to ’unknown’ in the improvement model, clinicians can more accurately communicate to patients about sites that are highly likely to respond positively to treatment, while avoiding the premature categorization of the other sites as non-responding. This approach might not only help to manage realistic patient expectations but might also boost motivation toward a positive treatment outcome. The influence of prediction models on patients and therapy outcomes remains understudied and should be investigated in the future by prediction model impact studies^29^. In conclusion, while routinely collected baseline clinical data have been associated with periodontal therapy outcomes before, their predictive potential at the site level remains relatively limited. However, incorporating these data into machine learning models enhances predictive discrimination and lays the foundation for more advanced modeling alongside behavioral, environmental, genetic, and immunologic factors. Such models could prove valuable in patient education and communication, helping to improve motivation and set realistic expectations for immediate therapy outcomes.

## Methods

This study follows the TRIPOD^30^ guidelines, which are designed to enhance transparency and quality in the reporting of predictive modeling for medical prognosis.

### Sources of data and participants

Two sources of data were used for model development and validation. The main dataset was collected in the undergraduate clinical course of the Department of Conservative Dentistry and Periodontology of the LMU Hospital Munich in a retrospective clinical trial between February 2011 and March 2016, as described in detail previously^9^. Pre- and post-treatment measurements were performed by the same individual and were supervised, controlled, and corrected by two calibrated experienced dentists with an interrater reliability of 0.82^31^. It comprises 746 patients with 100,950 measured tooth-sites.

The validation dataset was collected retrospectively in the Department of Periodontology at the University of Leuven in secondary care. It comprised 94 patients with 13,750 measured tooth-sites, all collected by a single experienced dentist.

Only patients at least 18 years of age with a diagnosis of periodontitis according to the current classification were included in the study^32^. A full dental and periodontal charting was required for all participants, including probing pocket depth measurements at six sites per tooth, recorded at baseline (T0) and reevaluation (T1). Patients with periodontal therapy in their medical history two years prior to the study were excluded. Additionally, pregnant women and participants rejecting consent to data collection were also excluded. This resulted in seven participants excluded from the primary dataset and none from the external validation dataset. The study was conducted in accordance with the Declaration of Helsinki. All patients provided informed consent for data collection. Ethical approval for data collection was granted by the Ethics Committee of the Medical Faculty of the Ludwig-Maximilians-University, Munich (Reference No. 22-0669), and the Ethics Committee Research UZ/KU Leuven, Leuven (Reference No. S55431).

### Assessment of Periodontal Staging and Grading

To determine the severity of periodontitis in each patient and the distributions in each dataset, the stage, grade, and extent were calculated based on the current classification^32^. The required parameters were imputed from available measurements according to the ACES framework^33^. Clinical attachment loss (CAL) was estimated indirectly from baseline probing pocket depth (PPD) and baseline recession using

1 $${\mbox{CAL}}\,=\left\{\begin{array}{ll}{{\mbox{PPD}}}_{{{\rm{Baseline}}}}-3\,\,{\mbox{mm}}\,\qquad\qquad\quad\,{\mbox{if}}\,{{\mbox{Recession}}}_{{{\rm{Baseline}}}}=0\\ {{\mbox{PPD}}}_{{{\rm{Baseline}}}}+{{\mbox{Recession}}}_{{{\rm{Baseline}}}}\quad\,{\mbox{if}}\,{{\mbox{Recession}}}_{{{\rm{Baseline}}}} \,>\, 0,\end{array}\right.$$

where 3 mm represents the average physiological probing depth in healthy periodontal tissue.

Functional occlusion was estimated by counting the number of anatomical maxillary-mandibular tooth pairs where both corresponding teeth were present. Each maxillary tooth was considered to have a designated opposing mandibular tooth based on standard dental anatomy (e.g., tooth 16 opposes 46, 11 opposes 41). A pair was counted if both teeth were present in the same patient. Third molars were excluded. The total number of opposing tooth pairs per patient was calculated as:

2 $${n}_{{\rm{opposing}}}=\mathop{\sum}\limits_{i=1}^{N} {\mathbb{1}} \{ {\rm{both}}\, {\rm{teeth}}\, {\rm{in}} \,{\rm{pair}}\, i \, {\text{are}}\, {{present}}\},$$

where ${\mathbb{1}}\{\cdot\}$ denotes the indicator function, which equals 1 if the condition in the braces holds for a given *i*, and 0 otherwise and *N*=14 is the number of standard occluding pairs per patient, excluding third molars.

Alveolar bone loss was estimated based on a recent methodology for grading periodontitis in the absence of radiographic data^34^. Average root length values were obtained from Salonen et al.^35^ and mapped to each measurement according to the patient’s gender, tooth type, and interproximal site, yielding the following formula:

3 $$\,{\mbox{Bone loss}}\,\,[ \% ]=\frac{\,{\mbox{CAL}}}{{\mbox{Root length}}\,}\times 100.$$

Staging was based on the maximum interdental CAL and the number of opposing tooth pairs. Stage 1 was assigned if the maximum CAL was between 1–2 mm. Stage 2 was assigned for CAL between 3–4 mm, provided no additional evidence of disease progression was present. Stage 3 was assigned under two conditions: (i) if CAL was between 3–4 mm and either ≥2 non-adjacent teeth exhibited PPD ≥ 6 mm or FI was present (grade 2 or 3); or (ii) if CAL was ≥5 mm with at least ten occluding pairs. Stage 4 was assigned if CAL was ≥5 mm and fewer than ten occluding pairs were present, reflecting impaired masticatory function. The procedure is summarized as follows:

4 $$\,{\mbox{Stage}}\,=\left\{\begin{array}{ll}1,\quad {\mbox{if}}\,{\rm{max}}\,({\mbox{CAL}})\in [1,2]\\ 2,\quad {\mbox{if}}\,{\rm{max}}\, ({\mbox{CAL}})\in [3,4]\\ 3,\quad{\mbox{if}}\,{\rm{max}} \,({\mbox{CAL}})\in [3,4]\,{\mbox{and either}}\,\\ \qquad{\mbox{PPD}}\,\ge\, 6\,\,{\mbox{mm at}}\,\ge\, 2\,{\mbox{non-adjacent teeth,}}\,\\ \qquad{\mbox{or}}\,{\mbox{furcation involvement}}\,\in\, \{2,3\}\\ 3,\quad{\mbox{if}}\, ({\mbox{CAL}})\,\ge\,5\,{\mbox{and}}\,\,{n}_{{{\rm{opposing}}}}\ge 10\\ 4,\quad {\mbox{if}}\, ({\mbox{CAL}})\,\ge\,5\,{\mbox{and}}\,\,{n}_{{{\rm{opposing}}}}\,<\,10\end{array}\right.$$

Grades were determined using the ratio of bone loss to patient age and cigarette number per day. Diabetes status could not be incorporated due to the lack of HbA1c values:

5 $$\,{\mbox{Grade}}\,=\left\{\begin{array}{ll}\,{\mbox{A}}\,,\quad{\mbox{if}}\,\frac{{\mbox{Bone loss}}}{{\mbox{Age}}}\,<\, 0.25\\ \,{\mbox{B}}\,,\quad{\mbox{if}}\,0.25 < \frac{{\mbox{Bone loss}}}{{\mbox{Age}}}\,<\,1\,{\mbox{OR}}\,{\mbox{Cigarettes/day}}\,<\, 10\\{\mbox{C}}\,,\quad\,{\mbox{if}}\frac{{\mbox{Bone loss}}}{{\mbox{Age}}}\,>\,1\,{\mbox{OR}}\,{\mbox{Cigarettes/day}}\,>\, 10.\end{array}\right.$$

Extent was classified as localized or generalized based on the proportion of teeth at the maximum stage:

6 $${\rm{Extent}} [\%] = \frac{n_{{\text{max}}\,{\text{stage}}}}{n_{{\text{teeth}}\,{\text{present}}}} \times 100.$$

Patients were considered to have generalized periodontitis if this percentage exceeded 30%; otherwise, the disease was classified as localized.

### Therapy

All participants received step I and step II periodontal therapy as described previously^9^. After the diagnosis of periodontitis, every patient received step I periodontal therapy with in-detail information on the etiology, pathogenesis, risk factors, and treatment of the disease, followed by oral hygiene instructions and professional mechanical supragingival plaque removal. Afterwards, step II non-surgical antiinfective periodontal therapy was carried out on all teeth presenting with PPD > 3 under local anesthesia using manual curettes (Gracey SG5/6, SG7/8, SG 13/14, SG15/16)(Hu-Friedy, Chicago, USA), and sonic devices (Sonic Flex) (KaVo, Biberach, Germany) with working tips for subgingival instrumentation (No. 60/61/62) (KaVo) without restrictions to time or number of visits. Adjunctive systemic antibiotics were prescribed in cases of advanced periodontitis or when specific periodontal pathogens -particularly Aggregatibacter actinomycetemcomitans- were identified using a commercial microbial test. Patients meeting the clinical or microbiological indications received metronidazole 400 mg three times daily for 7 days. In cases where A. actinomycetemcomitans was detected, amoxicillin 500 mg three times daily was added to the regimen for the same duration. No other adjunctives were used during treatment. In the main dataset, each periodontal treatment was performed by a separate individual with a similar experience level. However, therapy was controlled on each periodontal site by the two calibrated supervising dentists with a clinical focus on periodontology. Non-surgical reinstrumentation as part of step III was not included in the analysis to ensure a discrete evaluation of initial step II therapy outcomes. However, outcomes have been reported previously^36^.

Therapy in the validation dataset followed a similar protocol. The only notable difference was that Step II non-surgical anti-infective periodontal therapy was always administered over two consecutive appointments. Additionally, patients were prescribed a 0.2% chlorhexidine mouth rinse (10 ml, twice daily for one minute) for a duration of seven weeks. After 8 weeks, patients returned for an oral hygiene control visit, during which their oral hygiene practices were assessed, adjusted if necessary, and reinforced through motivational guidance.

### Outcome

For all tasks, the outcome was based on PPD at reevaluation 6.33 ± 3.79 month (mean ± SD) after completion of step II therapy in the main dataset and 7.65 ± 4.3 month in the validation dataset. Three clinically relevant outcome parameters were analyzed. Multiclass classification models were used to classify PPD after therapy into three groups, i.e., ≤3 mm, PPD = 4–5 mm, or PPD ≥ 6 mm aimed at predicting the necessity for different follow-up treatments. Furthermore, binary classification models were trained to predict whether baseline-affected pockets (PPD ≥ 3) would improve or not improve after therapy. Lastly, binary classification models were trained on baseline-affected pockets (PPD ≥ 3) to predict pocket closure, i.e., PPD ≤3 or 4 mm without BOP after therapy^37^.

### Predictors

Predictors were selected across three hierarchical levels at patient-, tooth- and site-level based on both their clinical relevance and routine availability. All data were collected at T0 prior to the occurrence of the outcome. A table with predictor distributions and explanations can be found in the appendix (Supplementary Table 1, 2). Patient level data consisted of patient characteristics: age, sex, body mass index (BMI), antibiotic treatment, periodontal family history, orthodontic treatment history, diabetes, stress level (rated 1–10) as well as smoking habits (smoking-type and cigarette number) (Supplementary Table 1). Behavior data, such as stress level or smoking habits, were assessed through clinician-reported evaluations from structured interviews. Confounding factors on tooth level included FDI-notation (tooth), tooth type, root number, mobility, restoration, pulp vitality, percussion sensitivity and cold sensitivity (Supplementary Table 2). Tooth site, PPD, recession, BOP, plaque, and FI were included as predictors on site level (Supplementary Table 2). Experiments were either conducted with one-hot or target encoding categorical predictors.

### Missing data

Each predictor was assessed individually based on clinical considerations for data imputation. Patient characteristics, such as age or gender were recovered from clinical records. Missing values in BOP, percussion sensitivity, smoking type, restoration, baseline recession as well as diabetes were imputed as non-occurrence, while vitality was imputed as positive based on their clinical likelihood of non-notation. Stress level was imputed based on the overall median, and BMI on the overall mean. If individual plaque data points were missing, they were imputed as “no plaque.” For patients with entirely missing plaque data, imputation was based on the majority occurrence at similar teeth, corresponding sites, and comparable PPD. Root number and tooth type were inferred from the FDI-notation. Patients missing FI at some sites were imputed as having no FI at these sites. For patients missing all furcation data, values were imputed based on baseline PPD plus recession: total <3 mm = grade 0, 3–6 mm = grade 1, >6 mm = grade 2. All sites anatomically incapable of developing FI (i.e., single-rooted teeth) were recorded as having no FI

### Statistical analysis

Predictive modeling consists of four consecutive steps which were adapted specifically for periodontal therapy outcome prediction (Fig. 1A). A benchmarking pipeline was established to apply linear as well as non-linear predictive models with different complexities to the dataset (Fig. 1B). A LR was trained as a representative of a linear model. In LR, the influence of many predictors toward a single outcome is calculated by adjusting weights iteratively. LR models were trained using the saga solver, with log loss for binary tasks and a multinomial loss function for multiclass classification. An MLP is a fully-connected feedforward neural network that consists of an input layer, one or more hidden layers, and an output layer. Each neuron, in one layer, connects with a certain weight to every neuron in the next layer. MLPs can model non-linear relationships through neurons in the hidden layers, which apply a non-linear activation function to the weighted sum of their inputs^38^. An RF was chosen as a simple and XGBoost as a complex representation for tree-based predictive models. RF is an ensemble learner that constructs multiple decision trees in parallel during training and predicts the majority vote of the ensemble for classification problems^39^. In boosting, a series of base learners is built in a sequential manner, where each subsequent model aims to correct the errors made by the previous models. The core idea is to combine several weak learners that are only slightly better than random guessing into a strong learner that achieves significantly better performance^40^. For binary classification, XGBoost was employed with a log loss and for multi-class classification with a multi-class log loss. An 80-20 train-test split was applied as the resampling strategy. To prevent snooping bias, data were grouped at patient level, ensuring that each patient appears exclusively in either the train or test dataset without overlap. Hyperparameter were either optimized by random search or Heteroscadistic evolutionary Bayesian optimization (HEBO) with holdout or 10-fold cross-validation. To adjust for unbalanced classes, models were tuned on either F1 or Brier score for binary classification problems and either F1_macro_ or multiclass Brier score for multiclass classification.

The models were first validated internally using an independent test set from the train/test split. To improve performance, models were retrained on the average performance across multiple splits. A dummy classifier, which predicts solely based on majority class voting without incorporating any features, was used as a baseline to assess model predictive utility.

Baseline comparisons were conducted using untuned LR and RF models, trained with default hyperparameters and no additional optimization. Model performance was evaluated using multiple metrics: Accuracy, Precision, Recall, F1-Score, Brier Score, and Brier Skill Score, chosen to comprehensively assess classification effectiveness and reliability. Only the best-performing model from each tuning criterion was selected for in-detail analysis. Additionally, calibration plots were generated to analyze each model’s probability distribution and identify potential over- or underestimation biases. Afterwards, models were tested against the independent external dataset. To assess feature importance, SHapley Additive exPlanation (SHAP) was applied to quantify the contribution of each feature to a model’s predictions.

### Software

The predictive modeling pipeline was developed in Python (3.11) and has been published open access online "periomod". Statistical methods were established based on Scikit-learn (1.5.1)^41^, XGBoost (2.1.1)^42^, imbalanced-learn (0.12.3)^43^ and HEBO (0.3.5)^44^. Graphs were plotted in Matplotlib (3.9.2)^45^ and Seaborn (0.13.2)^46^. Feature importance was determined by SHAP (0.46.0)^47^. Periodontal chart data of the external dataset were automatically extracted in Python (3.11), which utilized the library pdfplumber (0.11.5) for parsing PDF files. Patient-level features were extracted manually from clinical records.

## Supplementary information

Supplementary information

## Data Availability

The data used in this study are not publicly available due to patient privacy concerns but are available from the corresponding author upon reasonable request.

## Code availability

The code has been incorporated into a software package “Periomod” and published open access online. The package is available at https://github.com/Tobias-Brock/periodontal-modeling^48^.
