# Supplementary material for: Predictive modeling for step II therapy response in periodontitis - model development and validation
Source: NPJ Digit Med. 2025 Jul 15;8:445. doi: 10.1038/s41746-025-01828-3 (PMC12264052; doi:10.1038/s41746-025-01828-3)
Supplement: Supplementary file 1 — Supplementary information [file 41746_2025_1828_MOESM1_ESM.pdf]

# – Supplementary Information –

Predictive Modeling for Step II Therapy Response in Periodontitis – Model Development and Validation

Elias Walter[1*], Tobias Brock[2], Pierre Lahoud[1,3,4], Nils Werner[1], Felix Czaja[2], Antonin Tichy[1], Caspar Bumm[1], Andreas Bender[2,5], Ana Castro[3], Wim Teughels[3], Falk Schwendicke[1], Matthias Folwaczny[1]

[1]Department of Conservative Dentistry and Periodontology, University Hospital, LMU Munich, Goethestraße 70, Munich, 80336, Bavaria, Germany.
[2]Department of Statistics, LMU Munich, Munich, 80799, Bavaria, Germany.
[3]Division of Periodontology & Oral Microbiology, Department of Oral Health Sciences, University Hospitals Leuven, KU Leuven, Leuven, 3000, Belgium.
[4]OMFS-IMPATH Research Group, Department of Imaging and Pathology, University Hospitals Leuven, KU Leuven, Leuven, 3000, Belgium.
[5]Munich Center for Machine Learning (MCML), Munich, Bavaria, Germany.

*Corresponding author(s). E-mail(s): Elias.Walter@med.uni-muenchen.de;

Supplementary Table 1: Distribution of stage, grade and extent across train, test, and validation datasets in absolute counts and percentages.

		n			%		
Type	Category	Train	Test	Val	Train	Test	Val
Stage	0	2	1	0	0.3	0.7	0.0
	1	29	9	0	4.9	6.1	0.0
	2	34	8	0	5.8	5.4	0.0
	3	295	63	68	49.9	42.6	73.9
	4	231	67	24	39.1	45.3	26.1
Grade	A	17	4	0	2.9	2.7	0.0
	B	252	54	29	42.6	36.5	31.5
	C	322	90	63	54.5	60.8	68.5
Extent	Localized	336	97	2	56.9	65.5	2.2
	Generalized	255	51	90	43.1	34.5	97.8
Included Patients	Total	591	148	92	100	100	100

Supplementary Table 2: Class distribution of patient level features.

Level	Feature	Category / Unit	Value
Patient-level	Gender	Male	348
		Female	393
	Pregnant	No	414
		Yes	3
	Periodontal Family History	No	236
		Unknown	246
		Yes	186
	Smoking Type	No	339
		Cigarette	113
		Pipe	7
		Cigar	6
		All	1
	Diabetes	No	670
		Type I	13
		Type II	31
		Type II - drug induced	26
	Antibiotic Treatment	Yes	109
		No	637
	Age	years	56 ± 14
	Body Mass Index	kg/m^2	26 ± 4
	Cigarette Number	cigarettes/day	13 ± 8
	Stresslevel	0-10	5 ± 3

Supplementary Table 3: Class distribution of tooth- and site-level features.

Level	Feature	Category / Mean Value	Value
Tooth-level	Mobility	Yes	2686
		No	14139
	Tooth type	Anterior	7909
		Premolar	4626
		Molar	4290
	Root number	Singlerooted	11445
		Multirooted	5380
	Restoration	None	8109
		Filling	4711
		Crown	4005
	Percussion-sensitivity	No	16495
		Yes	293
	Sensitivity	Positive	14324
		Negative	2449
Site-level	Site	Distal-vestibular	16825
		Vestibular	16825
		Mesial-vestibular	16825
		Mesial-oral	16825
		Oral	16825
		Distal-oral	16825
	PPD Groups	≤ 3	80606
		$3-5$	14737
		≥ 6	5607
	Furcation Baseline	No furcation involvement	8123
		Furcation palpable	2456
		1 - 3 mm Furcation	1003
		$\geq$ 3mm Furcation	179
	BOP	Negative	63378
		Positive	36939
	Plaque	Negative	60515
		Positive	37922

Supplementary Table 4: Descriptive analysis of missing datapoints.

Level	Feature	Missing Value Count	[%]
Patient-level	Age	0	0.0
	Gender	0	0.0
	Pregnant	329	44.1
	Perio family history	78	10.5
	Smoking type	280	37.5
	Diabetes	6	0.8
	Antibiotic treatment	0	0.0
	Body mass index	12	2.0
	Cigarette number	13	10.0
	Stresslevel	19	3.0
Tooth-level	Mobility	0	0.0
	Toothtype	0	0.0
	Rootnumber	0	0.0
	Restoration	0	0.0
	Percussion-sensitivity	37	0.2
	Sensitivity	52	0.3
Site-level	Site	0	0.0
	PPD baseline	0	0.0
	Recession baseline	9878	10.0
	Furcation baseline	1086	8.5
	BOP	633	0.6
	Plaque	2513	2.5
